# Administration of Caffeine in Alternate Forms

**DOI:** 10.1007/s40279-017-0848-2

**Published:** 2018-01-24

**Authors:** Kate A. Wickham, Lawrence L. Spriet

**Affiliations:** Human Health and Nutritional Sciences, University of Guelph, Guelph, ON N1G 2W1 Canada

## Abstract

There has been recent interest in the ergogenic effects of caffeine delivered in low doses (~ 200 mg or ~ 3 mg/kg body mass) and administered in forms other than capsules, coffee and sports drinks, including chewing gum, bars, gels, mouth rinses, energy drinks and aerosols. Caffeinated chewing gum is absorbed quicker through the buccal mucosa compared with capsule delivery and absorption in the gut, although total caffeine absorption over time is not different. Rapid absorption may be important in many sporting situations. Caffeinated chewing gum improved endurance cycling performance, and there is limited evidence that repeated sprint cycling and power production may also be improved. Mouth rinsing with caffeine may stimulate nerves with direct links to the brain, in addition to caffeine absorption in the mouth. However, caffeine mouth rinsing has not been shown to have significant effects on cognitive performance. Delivering caffeine with mouth rinsing improved short-duration, high-intensity, repeated sprinting in normal and depleted glycogen states, while the majority of the literature indicates no ergogenic effect on aerobic exercise performance, and resistance exercise has not been adequately studied. Studies with caffeinated energy drinks have generally not examined the individual effects of caffeine on performance, making conclusions about this form of caffeine delivery impossible. Caffeinated aerosol mouth and nasal sprays may stimulate nerves with direct brain connections and enter the blood via mucosal and pulmonary absorption, although little support exists for caffeine delivered in this manner. Overall, more research is needed examining alternate forms of caffeine delivery including direct measures of brain activation and entry of caffeine into the blood, as well as more studies examining trained athletes and female subjects.

## Introduction

Caffeine is a socially acceptable drug that has been used as an ergogenic aid or performance enhancer in athletic circles for many years. It is a currently legal method of enhancing performance in training sessions and athletic competitions as it does not appear on the World Anti-Doping Agency’s banned or restricted substances list. Over the last 10 years, numerous reviews have examined different aspects of the efficacy of caffeine as an ergogenic aid [1–6] and a book was published to “describe a framework that might help the world of sport to develop a sensible and unified view of caffeine use by athletes” [7]. The contemporary approach is to use low doses of caffeine which exert ergogenic effects through interactions with the central nervous system (CNS) and have minimal effects on the physiological responses to exercise and caffeine-related side effects [6].

The traditional form of caffeine administration in research and athletic settings has been to ingest tablets/capsules along with water or to drink coffee. The caffeine is quickly swallowed and the majority absorbed into the blood from the intestine, with the possibility that a small amount is absorbed in the buccal mucosa. Caffeinated sports drinks have also been studied for many years, with most reports demonstrating that caffeine added to a sports drink has a further performance enhancing effect above that of a carbohydrate (CHO)-electrolyte solution alone, as reviewed by Kovacs et al. [8], Cureton et al. [9], and Spriet [6]. These findings will not be reviewed here, but include studies that examined the effects of caffeinated sports drinks on cycling [10–12], running [13], golf [14] and soccer [15, 16] performance.

Caffeine is now also available in gels, bars, gums, lozenges and energy drinks, which may affect how quickly the caffeine is absorbed into the blood from the buccal mucosa and intestines. There is also recent evidence that mouth rinsing with caffeine may activate sensors in the oral cavity with direct connections to the brain that could ultimately affect athletic performance. Lastly, manufacturers are also suggesting that the delivery of caffeine in mouth and nasal aerosol sprays may activate sensors with neural links in the nose and provide a direct route for absorption in the lungs, although no research has examined this possibility. Given the interest in these so-called “alternate forms of delivery,” this paper aims to examine (1) how they affect the rate of caffeine entry into the blood versus traditional tablet or coffee administration, (2) if they stimulate direct connections between caffeine sensors in the oral and nasal cavities and the brain, and (3) if they are ergogenic in training and competition situations.

## Caffeinated Bars and Gels

Over the last decade, few studies have explored the potential ergogenic effects of caffeinated bars and gels. To date, only Hogervorst et al. [17] have tested the effects of repeated dosing with caffeinated energy bars on cycling performance and a battery of cognitive tests in 24 trained men. The subjects completed 150 min of submaximal cycling at 60% maximal oxygen consumption (VO_2max_) followed by a 5-min rest, and a time to exhaustion protocol at 75% VO_2max_. Additionally, the subjects underwent a cognitive battery at baseline, at 70 and 140 min into the submaximal cycle, and again at exhaustion. The conditions were a caffeinated bar with 100 mg caffeine and 45 g CHO, a non-caffeinated bar with 45 g CHO, or 300 mL of a non-caloric placebo beverage, administered immediately before and at 55 and 115 min into the submaximal cycling protocol. The cognitive battery assessed complex cognitive function through a Stroop test, a rapid visual information processing (RVIP) task and a visual search test, and simple cognitive function through an immediate recall task. Saliva samples were collected at baseline and immediately following exhaustion for determination of caffeine concentrations. Supplementation with a caffeinated bar increased salivary caffeine (5.93 μg/mL) compared to baseline (0.25 μg/mL) (ratio of salivary to plasma concentrations = 0.74 ± 0.08 [18]). Supplementation with caffeinated bars improved reaction time on the Stroop test and RVIP test during steady-state exercise and following exhaustion and improved speed and accuracy on a visual search test at the end of exhaustive exercise compared to the other two conditions. The caffeinated bars also improved time to exhaustion (1600 s) versus the non-caffeinated CHO bars (1150 s) and the placebo beverage (850 s) [17].

Surprisingly, only two studies have explored the effects of caffeinated gels on athletic performance. Cooper et al. [19] investigated the effects of repeated dosing with caffeinated gels on performance of four blocks of an intermittent sprint test (IST) in 12 recreationally active males. The participants consumed either a CHO (25 g), CHO (25 g) and caffeine (100 mg), or placebo gel 1 h prior to the first IST block, immediately prior to the first IST block and at the end of the second IST block. The authors reported no significant difference between the conditions for best sprint time, but there was a trend for faster sprint performance in the CHO and caffeine group when compared with the CHO-only and placebo gel groups. Additionally, following the third block of sprints, the CHO and caffeine group demonstrated a significantly decreased fatigue index and a lower rating of perceived exertion compared to the CHO-only and placebo gel groups. In a second study, Scott et al. [20] demonstrated that ingestion of a CHO (21.6 g) and caffeine gel (100 mg), 10 min before a 2000-m rowing task, significantly improved performance compared to a CHO-only gel in 13 male collegiate athletes (CHO 471 s vs. CHO/caffeine 466 s).

Taken together, these studies suggest that bars and gels with 100 mg caffeine improved cognitive function, time to exhaustion, and time trial (TT) performance. Lacking from these studies were plasma caffeine measurements, although it could be assumed that increases would mimic the findings from caffeine tablet and coffee consumption. More research in this area is needed as caffeinated bars and gels are key caffeine sources for athletes during training and competition, and there is presently no work examining female subjects.

## Caffeinated Chewing Gum

Much of the important early work with the delivery of caffeine in chewing gum was conducted with a military purpose. Studies had demonstrated the ability of caffeine delivered in capsules to reverse the prolonged wakefulness-induced decrements in alertness, mood and performance [21–23]. However, there is a time delay of 20–30 min before significant amounts of caffeine leave the gut, reach the blood and affect the CNS. Therefore, in military settings where it is important to restore alertness and performance as quickly as possible, it was hypothesized that delivering caffeine in a chewing gum may speed the rate of caffeine delivery to the blood by absorption through the buccal mucosa as well as the gut [24]. Absorption of drugs other than caffeine in a gum form had been demonstrated to be more rapid through the buccal cavity, in part because of the extensive vascularization in this region [24, 25]. In addition, absorption through the buccal mucosa/cavity avoids the first-pass metabolism which may occur in the intestines or liver when absorbed through the gut. Therefore, any increase in the rate of caffeine absorption with gum could lead to a faster biological effect in the body.

**Fig. 1:**
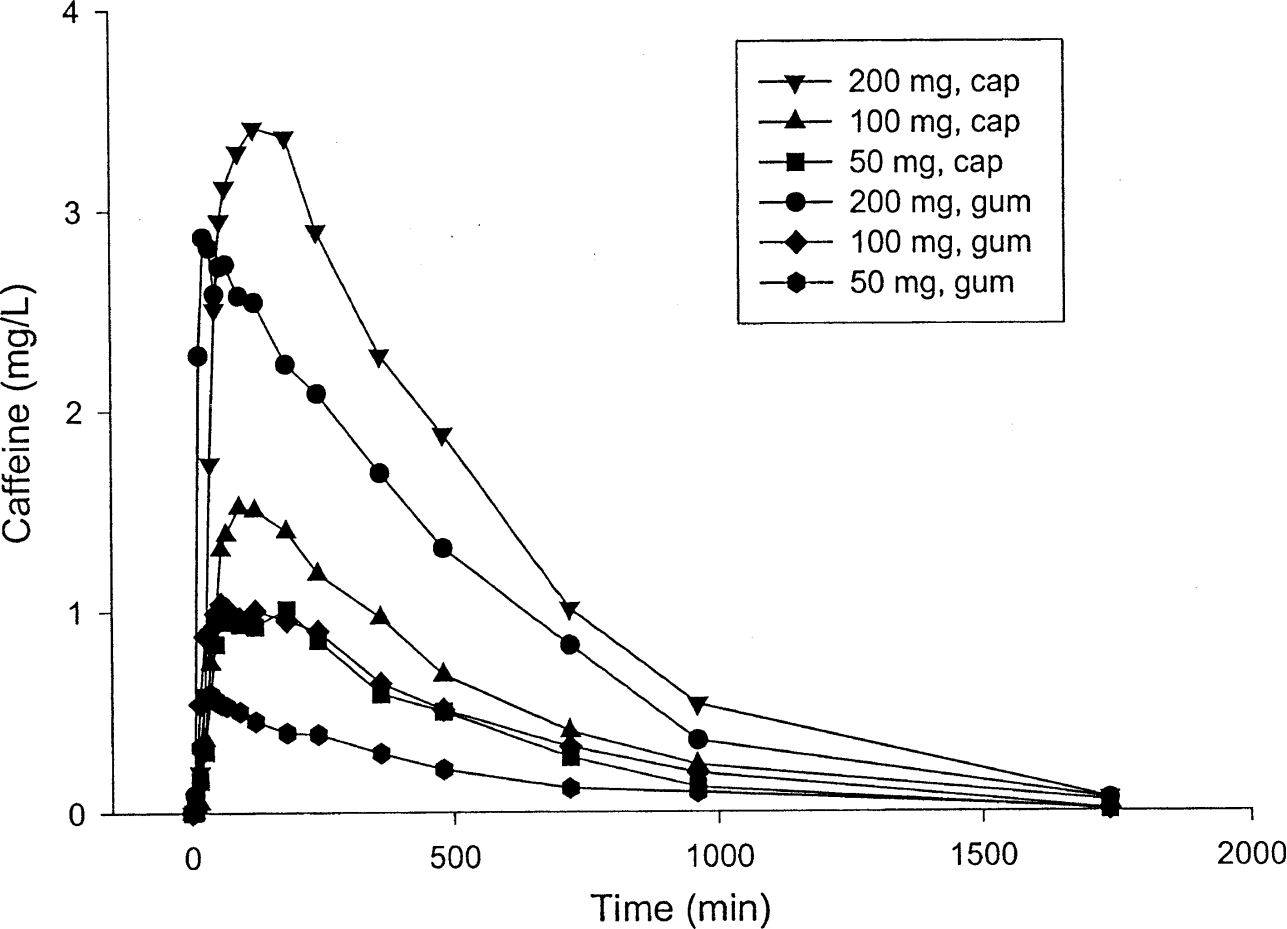
Mean caffeine plasma concentration profiles following a 50-, 100- or 200-mg dose of caffeine, delivered as either a capsule or gum formulation to healthy male volunteers (12 subjects in each of the seven treatment groups) Reproduced from Kamimori et al. [24], with permission

**Fig. 2:**
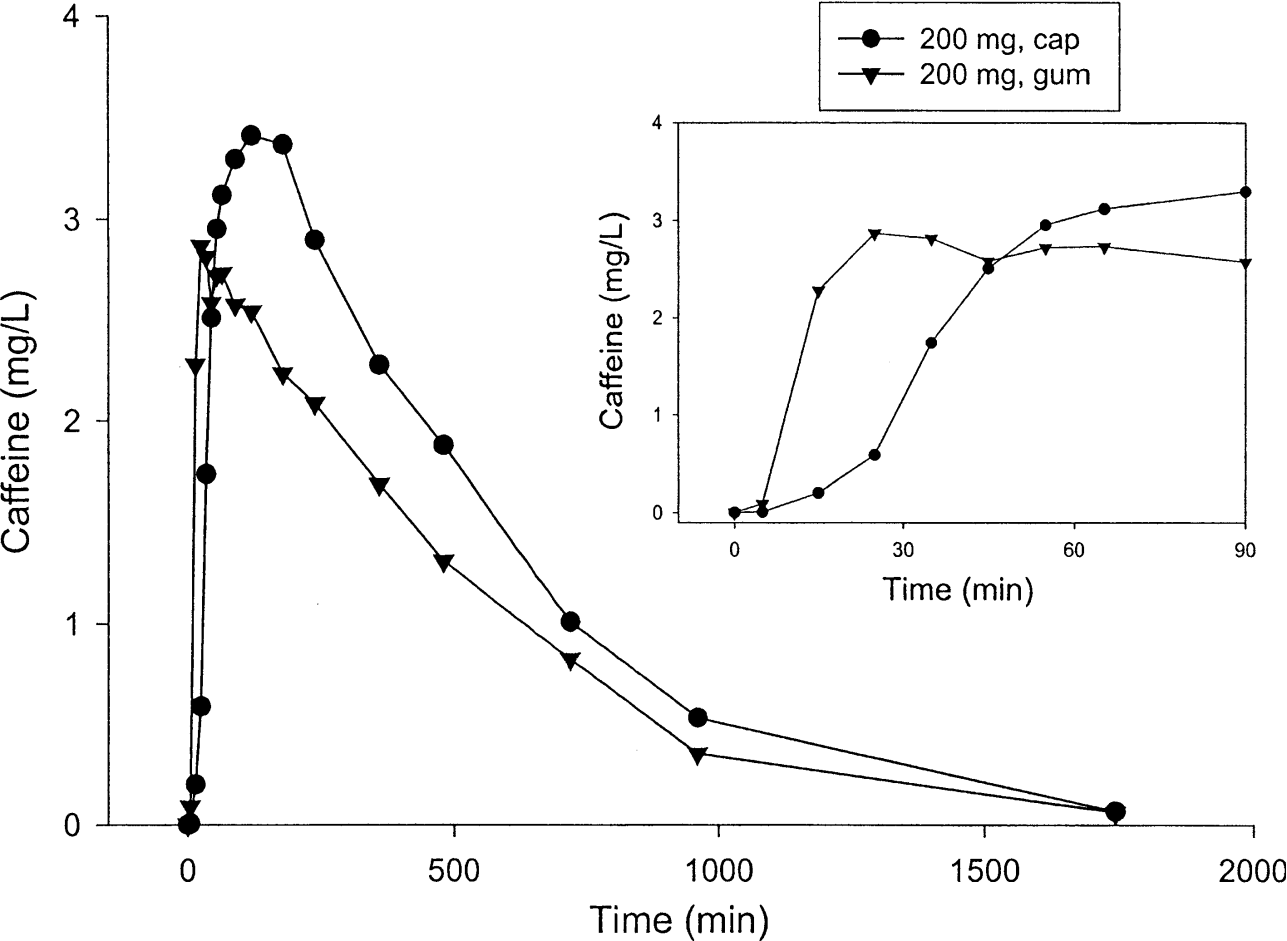
Mean caffeine plasma concentration profiles following a 200-mg dose of caffeine as a capsule or gum formulation to healthy male volunteers (12 subjects in each of the two treatment group). Inset shows plasma concentration profiles of the 200-mg dose delivered in capsule or gum formulation up to 90 min after caffeine administration Reproduced from Kamimori et al. [24], with permission

To test this hypothesis, a landmark study by Kamimori et al. [24] examined the rate of caffeine absorption by measuring plasma caffeine concentrations at several time points following the ingestion of capsules or chewing gum containing either 50, 100 or 200 mg of caffeine. Each condition had a separate group of 12 healthy male subjects who consumed less than 300 mg caffeine/day and had abstained from caffeine intake for 20 h and fasted for 3 h. Blood samples were taken at 5, 15, 25, 35, 45, 55, 65 and 90 min and 2, 3, 4, 6, 8, 12, 16 and 29 h post ingestion/chewing. The time to reach the maximal caffeine concentration was faster in the gum trials (44.2–80.4 min) versus the capsule trials (84–120 min). However, the maximal caffeine concentrations between capsule and gum conditions and the area under the entire concentration–time curves were not different at each of the three doses (Fig. 1). The markedly faster rate of absorption with the gum is seen when examining the 200-mg dose, as a large increase in plasma caffeine concentration occurred between 5 and 15 min and to a lesser extent from 15 to 25 min (Fig. 2). The largest increases in caffeine concentration with the capsules were delayed until 25–35 and 35–45 min. This study demonstrated the efficacy of delivering caffeine more quickly with gum versus capsules, in part by uptake in the buccal cavity along with absorption from swallowing while chewing gum. A second study from the same group demonstrated that plasma caffeine levels were maintained and increased in a dose-dependent manner with three repeated caffeine doses, each 2 h apart, when delivered in gum form with either 50, 100 and 200 mg of caffeine [26]. These pharmacokinetic findings are useful in military and sport situations where rapid caffeine effects are required and need to be maintained over a known time span. It is also possible that chewing gum may have an additional advantage over capsule delivery during intense exercise where splanchnic blood flow may be reduced and slow the absorption of caffeine in the gut, but this has not been studied to date.

### Caffeinated Gum and Athletic Performance

#### Aerobic Endurance Cycling

**Fig. 3:**
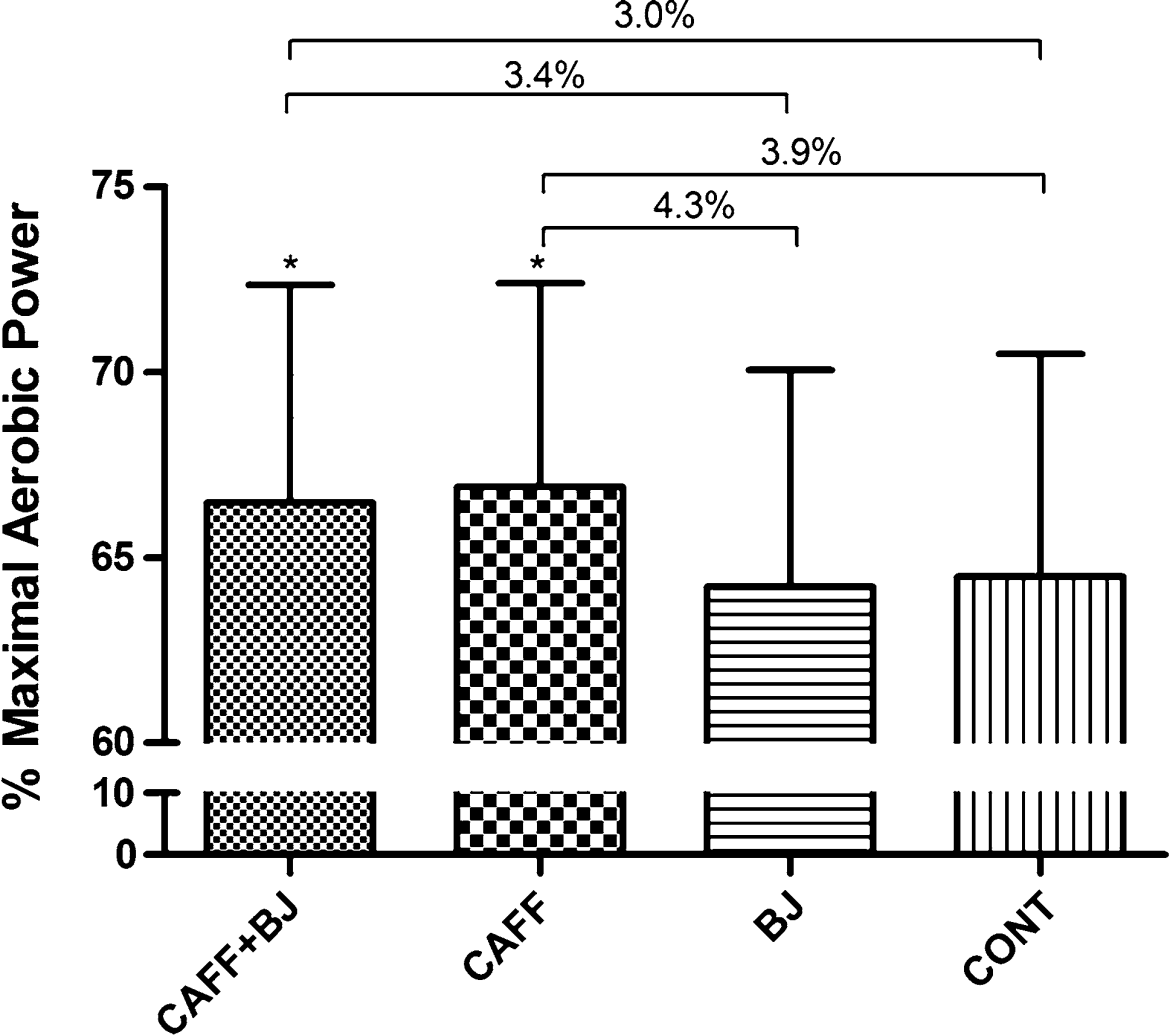
Mean power output combined for males and females during cycling time trial. Data are presented as mean ± standard deviation. *BJ* beetroot juice, *CAFF* caffeine, *CAFF* + *BJ* caffeine with beetroot juice, *CONT* placebo. *Different from CONT and BJ (*p* < 0.01) Reproduced from Lane et al. [29], with permission

Several studies have now examined the potential ergogenic effect of caffeinated gum administration on aerobic-based cycling. Ryan et al. [27] administered two sticks of caffeinated chewing gum (200 mg total) to college-age, physically active males at either 35 or 5 min before exercise, or 15 min into cycling at 85% VO_2max_ to exhaustion (~30–35 min). A placebo was given at the other two time points and all three points during the control trial. The caffeinated gum did not improve endurance performance at any of the administration times [27]. In a follow-up study, Ryan et al. [28] gave caffeinated gum (300 mg) or non-caffeinated gum to well-trained male cyclists at either 120, 60 or 5 min before cycling at 75% VO_2max_ for 15 min, followed by a TT where 7 kJ/kg body mass (BM) of work was completed as fast as possible. Caffeine improved cycling TT performance only in the trial where the caffeine was administered 5 min before exercise [28]. Lane et al. [29] examined the effects of 3 mg/kg BM of caffeine delivered in chewing gum to 12 well-trained males and 12 well-trained females during a TT that simulated the cycling course at the 2012 London Olympic Games (females 29.35 km, males 43.83 km), lasting 50–60 min. The athletes chewed caffeinated gum with 2 mg/kg BM for 10 min, starting at 40 min before the TT, and another 1 mg/kg BM in the 10 min before the TT. In the placebo trial, subjects chewed non-caffeinated gum. The subjects also underwent two additional trials, one with beetroot juice (BRJ) and one with BRJ and caffeine. The results were similar for females and males, and caffeine ingestion in the caffeine trial alone and in the caffeine + BRJ trial significantly improved TT performance by 3–4% versus placebo (Fig. 3). BRJ did not affect performance.

Oberlin-Brown et al. [30] had 11 well-trained male cyclists ride for 90 min at 63% VO_2max_, followed by a 20-km TT on four occasions—to test placebo, caffeine, CHO, and caffeine with CHO. The caffeine was administered in 50-mg sticks of gum at the start of the TT and at the completion of 5, 10 and 15 km for a total dose of 200 mg (2.7 mg/kg BM). There were no significant differences in TT performance between conditions, with all times between 32:20 and 32:27 min:s. It is possible that the use of small 50-mg caffeine doses administered only at the start of the TT and every ~ 8 min thereafter limited the ergogenic effect of caffeine in this study. Paton et al. [31] studied the effects of administering caffeinated (200–300 mg) or non-caffeinated gum at the 10-km mark of a 30-km TT in ten well-trained female and ten well-trained male cyclists. There also was a 0.2-km sprint (~ 15 s) at the end of each 10-km section. There were no differences in performance during the initial 20 km of the TT, but caffeine improved mean power by 3.8 ± 2.3% and increased speed by 1.9% in the final 10 km and improved sprint power by 4.0 ± 3.6% during the final sprint. Females and males increased mean power over the final 10 km by 4.3 ± 3.4 and 3.2 ± 3.0% and increased sprint time by 1.9 ± 5.0 and 6.2 ± 5.2%, respectively.

These studies suggest that caffeine delivered in chewing gum in a dose of ~ 200–300 mg is ergogenic in well-trained females and male cyclists when delivered prior to or during an endurance event. However, it should be noted that no study has compared the effects of chewing gum versus the traditional caffeine capsule ingestion on aerobic performance in the same group of subjects.

#### Sprint Cycling and Power Events

Paton et al. [32] gave caffeinated chewing gum to nine competitive male cyclists who completed four sets of 30-s maximal sprints (with 30 s of active recovery at 100 W), with five sprints/set. Subjects cycled for 5 min at 100 W following sets 1 and 3. Following set 2, subjects cycled for 10 min at 100 W and caffeinated (240 mg/3 mg/kg BM) or placebo gum was administered. The rate of power output decline in sets 3 and 4 (ten sprints) was significantly reduced by the caffeinated gum versus placebo. A second study reported that standing shot-put performance was improved following the administration of 100 mg caffeine in chewing gum in nine collegiate shot-put athletes [33]. The subjects chewed the gum immediately before attempting six throws (with 1 min between throws), and the performance of the first throw and the overall performance of all six shot-put throws was improved with caffeine. Although this study utilized a small sample size, the results suggested that caffeinated gum improved performance in sprint and power events.

There was also one study that assessed the ergogenic effects of a caffeinated lozenge, and while this is not gum, the lozenge is held in the mouth for several minutes [34]. The lozenge contained 420 mg of nitric oxide (NO) and 70 mg of caffeine compared to a non-caloric placebo lozenge. The treatment was administered to 15 moderately trained cyclists (eight males, seven females) 10 min prior to the beginning of a cycling protocol where subjects cycled for 8 min at 50%, 6 min at 65% and 6 min at 75% VO_2max_, and then rested for 5 min before completing a 21.15-km TT. TT performance was significantly faster (2.1%) with the caffeinated lozenge (2424 s) compared to placebo (2477 s). In this study, the authors could not distinguish between the effects of NO and caffeine, and therefore could not be certain that caffeine was the only active ingredient [34].

## Caffeine Mouth Rinsing

Caffeine mouth rinsing is a relatively new form of caffeine supplementation. This modality gained traction alongside the emerging interest associated with the potential ergogenic effects of CHO mouth rinsing [35, 36]. It was originally proposed that caffeine mouth rinsing for 5–20 s elicited its ergogenic effects by allowing caffeine molecules to competitively inhibit adenosine through binding to adenosine receptors located in the mouth [37, 38]. This interaction was thought to increase permeability of the buccal mucosa therefore triggering caffeine absorption into the blood stream [39]. However, the time for this to occur would be short and the only study examining caffeine mouth rinsing that measured blood caffeine concentrations reported no increase in blood caffeine concentrations [40]. Evidently, a more feasible mechanism of action has been proposed to explain the performance benefits associated with caffeine mouth rinsing. The oral cavity is decorated with bitter taste receptor cells specifically located in the oropharyngeal epithelia [41], and these have been shown to be activated when exposed to caffeine [42]. It has been proposed that activation of these bitter taste receptors can activate gustatory neural pathways [41] and ultimately stimulate regions of the brain associated with information processing and reward [43, 44]. These same regions are shown to be activated when participants are administered a CHO mouth rinse [36, 43].

### Cognitive Performance

Using functional magnetic resonance imaging (fMRI), De Pauw and colleagues [45] identified in ten healthy males that caffeine mouth rinsing increased activity in the dorsolateral prefrontal cortex and the orbitofrontal cortex, which are brain regions associated with problem solving and reward, respectively. Furthermore, this group demonstrated that caffeine (1.2%) mouth rinsing, when administered as a 25-mL solution for 20 s, improved reaction time on an incongruent Stroop task (where the color of the word and the meaning do not match) compared to a CHO (6.4%) mouth rinse, and a placebo rinse. There was no significant difference on incongruent Stroop task performance between the CHO and placebo conditions [45]. Pomportes et al. [46] tested the effects of caffeine (67 mg), CHO (7%), and guarana (0.4 g) mouth rinses on cognitive function during 40 min of submaximal cycling versus a placebo rinse in 24 physically active participants (16 males, six females). The subjects were instructed to mouth rinse with 25 mL of the treatment for 20 s immediately before cycling. After 1 min of cycling, the subjects completed a duration-production task (to assess time perception) lasting 3 min, continued to cycle for 7 min, then completed the Simon task (to assess cognitive control and information processing) lasting another 3 min. This cognitive battery was repeated two more times with a 20-s mouth rinse occurring between batteries. The results showed that mouth rinsing with caffeine, CHO, or guarana resulted in more consistent responses during the duration-production task compared to placebo and shorter production durations, meaning that participants underestimated the duration of the task compared to placebo. There were also no differences between the caffeine, CHO or guarana treatments for variability or production durations. These authors suggest that mouth rinsing with caffeine, CHO, or guarana may increase brain activation and arousal compared to placebo. Interestingly, the authors also noted a smaller difference between mean incongruent reaction time and mean congruent reaction time during the Simon task in the caffeine condition (24 ms) compared to placebo (30 ms), CHO (29 ms) and guarana (29 ms) conditions, indicating improved cognitive control. There were no differences in errors between conditions.

Although there is minimal evidence to support the effects of caffeine mouth rinsing on cognitive performance, the evidence presented here suggests there may be a beneficial effect on reaction time and cognitive control. However, additional work is required with direct measures of brain activation and plasma caffeine concentrations.

### High-Intensity Repeated Cycle Sprinting

Beaven et al. [37] investigated the effects of caffeine mouth rinsing on repeated sprint cycling performance in 12 recreationally active males. The first experiment compared the effects of a 6% CHO mouth rinse solution, a 1.2% caffeine rinse solution, and a placebo rinse. The subjects completed a 5-min warm up before swirling 25 mL of the rinse solution around their mouths for 5 s and then expelling the solution. Immediately following the mouth rinse, subjects completed a 6-s all out sprint against a resistance equal to 10% of their BM. The subjects then received a 24-s rest period in which they were instructed to mouth rinse again for 5 s. The 6-s sprint and subsequent rest period with mouth rinsing was repeated a total of five times. The authors found that caffeine and CHO mouth rinses improved mean power in the first sprint compared to placebo. Furthermore, 50% of the participants elicited their greatest maximal power during the first two sprints in the caffeine mouth rinse condition when compared to the CHO and placebo rinses [37]. In the second experiment, the authors investigated the effects of a combined caffeine and CHO mouth rinse versus a CHO-only mouth rinse using the same exercise protocol as the first experiment. The combined caffeine and CHO mouth rinse elicited an increase in peak power during the first sprint and increased mean power during the last sprint compared to the CHO only rinse.

**Fig. 4:**
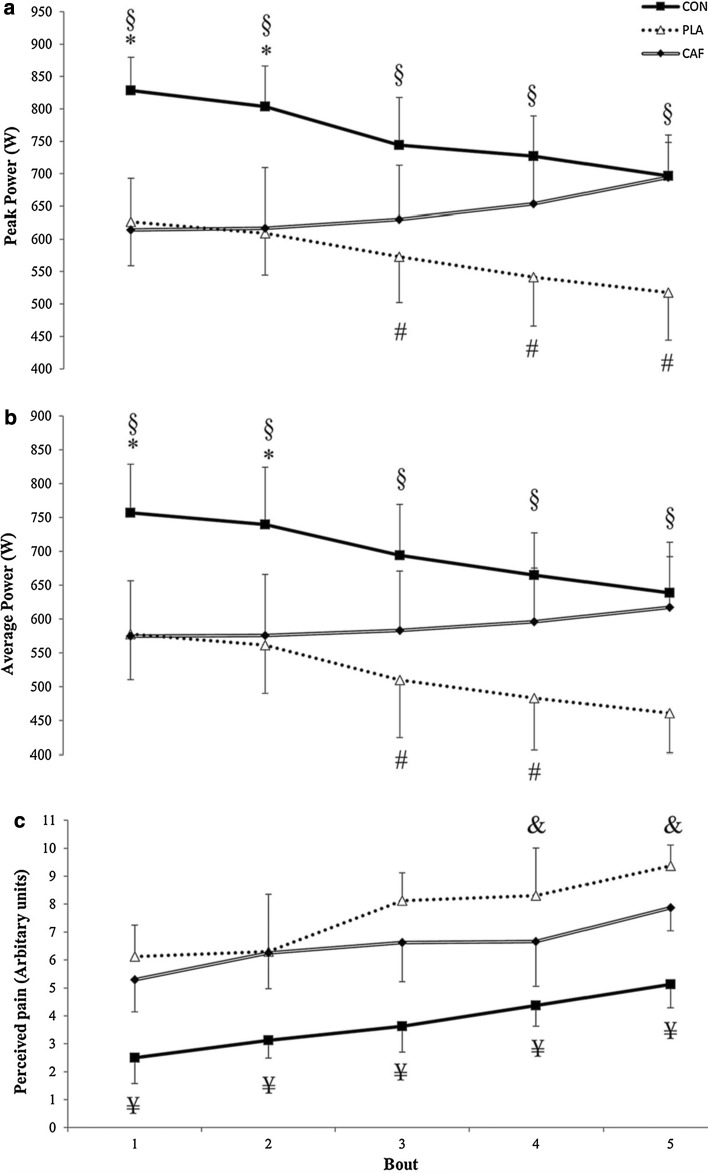
Peak and average power profiles and ratings of perceived pain for five, 6-s sprints separated by 24 s active rest in control (CON), glycogen depletion and placebo (PLA), and glycogen depletion and caffeine (CAF) conditions. **a** Peak power; **b** mean power; and **c** perceived pain. Data are presented as mean ± standard deviation. ^§^CON significantly greater than PLA (*p* < 0.05); # CON significantly greater than CAF (*p* < 0.05); ^#^CAF significantly greater than PLA (*p* < 0.05); ^¥^CON significantly less than PLA (*p* < 0.05); ^&^CAF significantly less than PLA (*p* < 0.05) Reproduced from Kizzi et al. [47], with permission

Kizzi et al. [47] employed the same exercise protocol as Beaven et al. [37], but induced a state of glycogen depletion (estimated at 30% of resting glycogen levels) prior to the repeated sprint protocol. This group explored the effects of mouth rinsing with 25 mL of a 2% caffeine solution versus a placebo rinse in a glycogen-depleted state in eight recreationally active males. The mouth rinse was performed for 10 s before the first sprint and in the rest periods between each subsequent sprint. The protocol was also repeated in a no-rinse, glycogen-rich state to provide a control group. As expected, the authors found that mean and peak power were highest in the control group for the first three sprints (Fig. 4). Furthermore, in the third sprint, mean and peak power were higher in the caffeine rinse group compared to placebo. Interestingly, there was no significant difference between the control group and the caffeine mouth rinse group for mean and peak power during sprints 4 and 5, and mean and peak power were significantly lower in the placebo group. Similarly, subjects’ perceived pain was lower for the first three sprints in the control condition, and perceived pain was lower in the caffeine condition compared to placebo during the third sprint [47]. There was no difference in perceived pain during sprints 4 and 5 when comparing the control condition to caffeine mouth rinse (Fig. 4). However, perceived pain was significantly higher in the placebo versus control and caffeine rinsing during sprints 4 and 5. It should be noted that no measures of muscle glycogen or plasma caffeine levels were made in this study.

It appears that short-duration, high-intensity, repeated-bout sprinting is improved with caffeine mouth rinsing in normal and glycogen-depleted states.

### Aerobic Exercise

The evidence surrounding the ergogenic effects of caffeine mouth rinsing and aerobic exercise performance is equivocal. Sinclair and Bottoms [48] investigated the effects of a tasteless 6.4% CHO mouth rinse, a tasteless 0.032% caffeine rinse, and a water rinse during a 30-min arm crank TT in 12 healthy males. The subjects rinsed with a 25-mL solution for 5 s immediately before starting the TT, and repeated the mouth rinse procedure every 6 min throughout the TT. The results indicated a greater distance covered during a 30-min arm crank TT when utilizing caffeine or CHO mouth rinses (~ 15 km) compared to a water rinse (~ 13 km). This greater distance was achieved by higher power outputs and revolutions per min in the caffeine and CHO rinse conditions.

Conversely, Doering et al. [40] found no effect of a caffeine mouth rinse in ten well-trained male cyclists performing a TT in which they had to complete the work equivalent to 75% of peak power output for 1 h (lasted ~ 65 min). These subjects were administered a 25-mL solution containing 35 mg of caffeine or a placebo rinse immediately before the TT and at 25, 50, 75 and 90% completion of the TT, and were instructed to rinse the solution around their mouths for 10 s before expelling the solution.

Pataky et al. [49] investigated the effects of caffeine capsule ingestion, caffeine mouth rinsing, and the combination on 3-km cycling TT performance in 38 recreationally trained cyclists (25 males, 13 females). This group also explored the effects of caffeine mouth rinsing on TT performance with respect to caffeine metabolizer genotype and time of day. It is important to note that the subjects were divided into two genotypes: AA homozygous (*n* = 21), who typically experience greater ergogenic effects with caffeine due to a quicker accumulation of caffeine metabolites, and AC heterozygotes (*n* = 17) [50]. To assess the effect of time of day on caffeine mouth rinsing and 3-km TT performance, 15 participants completed all of their trials before 10 a.m. and 23 subjects completed all of their trials after 10 p.m. The treatment conditions included a placebo capsule with a placebo 25-mL mouth rinse, a 6-mg/kg caffeine capsule with a 25-mL placebo rinse, a placebo capsule with a 25-mL caffeine rinse containing 300 mg of caffeine, and a 6-mg/kg caffeine capsule with a 25-mL caffeine mouth rinse containing 300 mg of caffeine. The capsule was ingested 1 h before the exercise protocol, and the mouth rinse was administered immediately before a 5-min warm up and again at the end of the warm up just before the TT.

These authors found a 3% improvement in 3-km cycling TT performance with caffeine capsule ingestion and in the caffeine capsule with mouth rinsing condition when compared to placebo and the caffeine rinse condition [49]. Since there was no benefit of caffeine mouth rinsing alone, it is suggested that the ergogenic effects were solely attributed to caffeine capsule ingestion. Interestingly, these researchers found a positive effect of caffeine mouth rinsing only when the exercise protocol was performed in the morning compared to the evening, suggesting a diurnal effect. Lastly, these authors found that caffeine capsule ingestion was ergogenic for AC heterozygous caffeine metabolizers, and caffeine rinse and capsule ingestion was likely ergogenic for both AA homozygous and AC heterozygous caffeine metabolizers. However, it was also found that caffeine mouth rinsing was possibly detrimental to performance in AA homozygous caffeine metabolizers. More work will be needed to confirm these findings. It is important to consider the possibility that caffeine administered in forms that avoid absorption in the gut and first pass metabolism, such as caffeinated gum, mouth rinsing, or aerosol sprays, may lead to more consistent responses across subjects as genetic variability in caffeine metabolism can account for some of the individual responses demonstrated in many caffeine studies.

Lesniak et al. [51] investigated the effects of a CHO mouth rinse, a caffeine rinse, and a combined CHO and caffeine rinse on TT performance in seven recreationally active females. Subjects completed the work equivalent to 60% of their maximum work rate for 1 h as fast as possible (TT lasted ~ 61 min). These authors found no differences between the conditions. However, there was no placebo group to determine if caffeine mouth rinsing improved performance over baseline. Dolan et al. [52] studied the effects of caffeine mouth rinsing on intermittent exercise performance in ten competitive college lacrosse players. These researchers utilized the Yo–Yo Intermittent Recovery Test to mimic stop-and-go sports performance. The participants were instructed to rinse their mouth with 25 mL of either a 6% CHO solution, a 1.2% caffeine solution, a combined CHO and caffeine solution, or a water rinse. There was also a no rinse condition. There were no significant differences in intermittent sport performance between any of the conditions [52].

Currently, most of the literature indicates no ergogenic effect of caffeine mouth rinsing for 5–20 s on aerobic exercise performance [40, 49, 51, 52]. The study by Sinclair and Bottoms [48] is the only study supporting a beneficial effect of caffeine mouth rinsing on aerobic exercise performance.

### Resistance Exercise

Clarke et al. [38] explored the effects of a caffeine mouth rinse (1.2%), a CHO rinse (6%), a combined caffeine and CHO mouth rinse, a placebo rinse, and a water rinse on resistance exercise performance in 15 recreationally resistance-trained males. The subjects were instructed to rinse 25 mL of the treatment solution around their mouth for 10 s immediately prior to performing a bench press at 60% of their 1 repetition maximum (RM) until failure. There were no significant differences in the total weight lifted between CHO mouth rinsing (~ 1100 kg), caffeine rinsing (~ 1100 kg), combined CHO and caffeine rinsing (~ 1050 kg), water rinsing (~ 1050 kg), or control conditions (~ 1050 kg). As this is the only study assessing the effects of caffeine mouth rinsing on resistance exercise performance, more research is needed.

### Mouth Rinse Summary

The only exercise situation where it has been shown that caffeine mouth rinsing is ergogenic is with short-duration, high-intensity, repeated-bout cycling protocols. Similarly, it seems that caffeine mouth rinsing may prove beneficial in states of glycogen depletion, and earlier in the day compared to later in the afternoon. Future research should examine if rinsing for a longer duration promotes absorption of caffeine through the buccal mucosa and measure the pharmacokinetics of plasma caffeine concentrations in these situations. More research is needed to examine the effects of caffeine mouth rinsing in females, as only one study investigated caffeine mouth rinsing in women [51], and also in trained subjects, as only two studies examined trained populations [40, 52].

## Caffeinated Energy Drinks

While energy drinks are not generally designed for use during sporting activities, they are used before, during and after physical activity [53]. The active ingredients in energy drinks are high levels of CHO (~ 10–12%) and moderate levels of caffeine (~ 80 mg caffeine/250 mL). There are also suggestions that taurine (1000 mg/250 mL) is an active ingredient, although little research support exists [54]. Energy drinks also contain many other ingredients. Over the past 2 decades numerous studies have examined the potential ergogenic effects of caffeinated energy drinks on athletic performance [55]. However, most of these studies did not assess the ergogenic effects of the individual ingredients in caffeinated energy drinks. This makes it impossible to assess the relative importance of each potential active ingredient to any ergogenic effects seen.

This review will discuss the three studies that attempted to examine the potential ergogenic effects of individual ingredients [56–58]. Geiss et al. [56] investigated the effects of 500 mL of Red Bull (160 mg caffeine, 2000 mg taurine, 10.5 g glucose) versus Red Bull with just the caffeine and glucose versus Red Bull with just glucose on cycling performance in ten endurance-trained males. However, there were no trials with just caffeine or just taurine. The exercise protocol consisted of 60 min at 70% VO_2max_ immediately followed by 50 W increases every 3 min until volitional exhaustion. The Red Bull beverage was administered halfway through the submaximal exercise. In addition, 24 h later, subjects returned to complete a cycling protocol starting at 50 W and increasing by 50 W every 3 min until volitional exhaustion. The subjects had a prolonged time to exhaustion in the taurine and caffeine condition (857.8 ± 236.4 s) compared to the caffeine and glucose condition (689 ± 92.35 s) and the glucose only condition (791.8 ± 188.52 s). Time to exhaustion in the exercise bout 24 h later was also significantly longer only with the drink that contained taurine. These authors suggested that taurine was the main ergogenic ingredient in Red Bull and that caffeine and glucose had no effect, as times to exhaustion were prolonged in the taurine condition compared to the taurine-free conditions. However, this study did not test the individual effects of caffeine or taurine. The results imply some synergistic effect of having taurine, glucose and caffeine in the same drink, as studies examining the effects of taurine alone on TT performance and incorporation into skeletal muscle have seen no effect [54, 59].

Kammerer et al. [58] improved on the previous work and recruited 14 male soldiers to test the effects of 250 mL of a placebo beverage, a caffeinated beverage (80 mg caffeine), a taurine beverage (1000 mg taurine), a caffeine and taurine beverage, and a commercially available energy drink (Red Bull: 27 g CHO, 80 mg caffeine, 1000 mg taurine) administered 45 min before three physical tests and two cognitive tests. The physical tests consisted of a VO_2max_ test where time to exhaustion was recorded, a maximum handgrip strength test using both right and left hands, and three vertical jumps. The participants completed a focused attention task in which they were required to point out the numbers 1–38 randomly allocated on a grid with different sized digits, and a digit span test to assess attention and immediate auditory memory. This test required participants to repeat strings of nine numbers in forward order and strings of eight numbers in reverse order. The results demonstrated no significant differences between conditions on any of the physical or cognitive tests, suggesting no ergogenic effect of caffeine, taurine, or the combination with glucose on aerobic capacity, handgrip strength, jump performance or cognitive performance.

A study by Eckerson et al. [60] assessed the effects of 500 mL of sugar-free Red Bull (160 mg caffeine, 2000 mg taurine), a sugar-free drink containing only caffeine (160 mg caffeine), and a placebo beverage on bench press strength and endurance in 17 physically active men. Subjects performed repetitions to failure at a weight equivalent to 70% of their 1 RM. The results indicated that sugar-free Red Bull (114.9 ± 16.2 kg) and the caffeinated sugar-free drink (115.1 ± 16.2 kg) had no significant effect on 1 RM compared to placebo (114.1 ± 5.5 kg) and no effect on muscular endurance during this test (sugar-free Red Bull 1164.1 ± 147.0 kg; caffeinated sugar-free drink 1173 ± 170.6 kg; placebo 1141.5 ± 193.4 kg). This study suggested there was no benefit of sugar-free Red Bull (caffeine and taurine) or a caffeinated sugar-free drink on resistance exercise performance.

The current literature does not support the ergogenic effects of caffeine supplementation administered in the form of energy drinks. However, there is a need for additional studies examining the effectiveness of the individual components of caffeinated energy drinks on performance.

## Caffeinated Nasal and Mouth Aerosol Sprays

Caffeine nasal and mouth sprays are the latest alternative method of caffeine supplementation. It has been reported that nasal administration of drugs may affect the brain through several mechanisms. First, it is possible that some of the drug enters the systemic circulation, ultimately reaching the brain and crossing the blood–brain barrier. The nasal epithelium is an extremely permeable membrane that allows molecules with a mass cut off lower than 1000 Da to rapidly access the brain via the blood stream [61]. Caffeine molecules could easily cross the nasal epithelium and ultimately affect the CNS through nasal spray delivery since they have a low molecular weight of 194 Da [62]. However, it could be argued that the time for this to occur is too short to have a meaningful impact. Secondly, the drug can be transported directly from the nasal cavity to the cerebrospinal fluid and brain tissue via intracellular axonal transport through the olfactory and trigeminal neural pathways [61, 63]. This method of delivery requires small molecules to travel along axons spanning from the nasal epithelium to the brain [63], but there is no information on the time course of this phenomenon. Thirdly, it has been shown that there are bitter taste receptors in the nasal cavity, akin to those found in the mouth [64]. It is possible that caffeine nasal sprays can activate bitter taste receptors located in the nasal cavity, which form connections with the trigeminal nerve and ultimately stimulate regions of the brain associated with reward and information processing [64]. Lastly, aerosols could deliver caffeine directly to the lungs where absorption into the blood would be expected, thereby delivering caffeine directly to the heart. However, the exact mechanism(s) are not presently established.

The first study in this field examined the efficacy of caffeine and glucose nasal sprays in affecting brain activity and cognitive performance in ten healthy males [62]. Participants completed a Stroop task immediately before and after administering a nasal spray containing 15 mg/mL caffeine, a spray containing 80 mg/mL glucose, or a distilled water placebo spray. The nasal spray was dispensed twice in each nostril to optimally disperse the treatment, and was administered for a total duration of 20 s. These authors measured brain activity through electroencephalogram and event-related potential (P300). Interestingly, treatment with both the caffeine and glucose nasal sprays increased activation of the primary somatosensory cortex (receives and interprets touch), motor cortices (planning, execution and control of motor movements), dorsolateral prefrontal cortex (information processing and working memory), orbitofrontal cortex (information processing and decision making), posterior cingulate cortex (learning and motivation), insular cortex (emotional awareness), and supramarginal gyrus (language perception and processing) compared to the placebo nasal spray [62]. It is also important to note that treatment with a caffeine nasal spray also resulted in a significantly greater activation of the dorsolateral prefrontal cortex and orbitofrontal cortex than the glucose spray. The Stroop task is designed to test a subject’s information processing, decision making, and attention [65]. However, it is surprising that despite increasing the activation of these brain regions, there was no effect of caffeine nasal spray on cognitive efficiency as measured by P300 amplitude and latency during a Stroop task [62].

De Pauw et al. [66] performed a follow-up study on 11 moderately trained males, assessing the effects of a caffeine nasal spray, a glucose spray, or a placebo spray on Stroop task performance, Wingate sprint cycling performance, and a 30-min cycling TT. The Stroop task was performed before and after both exercise components and a 15-min rest occurred between exercises. Before each exercise test and at 25, 50 and 75% completion of the cycling TT, the subjects were administered a nasal spray containing either 15 mg/mL caffeine, 80 mg/mL glucose, or a placebo distilled water spray. The nasal spray was dispensed twice in each nostril for 20 s. Furthermore, these authors performed an additional trial to collect venous blood samples at baseline and 20 s after administering the caffeine nasal spray to measure plasma caffeine concentrations. There was no significant increase in blood caffeine concentrations 20 s after administration of the nasal caffeine spray, and it is not clear why serial samples were not taken. There was no effect of caffeine or glucose nasal sprays on mean or peak power output during the Wingate test (peak power 1069 W with placebo, 1046 W with glucose, 1082 W with caffeine). The caffeine nasal spray also had no effect on the 30-min cycling TT (caffeine 206 W, placebo 207 W). Lastly, caffeine and glucose nasal sprays had no impact on reaction time during the Stroop task compared to placebo at any time point throughout the protocol. These authors argued that the effects of a caffeine nasal spray on the brain may be too small to significantly improve exercise performance and/or the dose of caffeine may be too small to elicit an ergogenic effect.

There are few investigations of the efficacy of caffeine nasal sprays, and more work needs to be done to expand the literature in this area. More detailed measurements of plasma caffeine levels following repeated nasal spray doses could establish the efficacy of this procedure. If positive results were found, it is conceivable that caffeine nasal sprays could be applied to many exercise situations, most notably those that incorporate a large information-handling and cognitive component, such as stop-and-go team sports.

It is also important to mention the prevalence of caffeinated aerosols administered directly in the mouth and/or under the tongue. These products are readily on the market and are flaunted for their ability to “boost energy levels throughout the day”. Some of the most common products on the market include AeroShot Pure Energy, which claims to deliver 100 mg caffeine/spray [67], Instavit Instant Energy, which claims to deliver 30 mg caffeine/four sprays [68], or Primer Caffeinated Breath Spray, which claims to deliver 33 mg caffeine/spray [69]. However, there is no current research examining these claims. Additionally, similar to caffeine nasal sprays, there is some concern about the safety of these products. If caffeine is administered as an aerosol in much larger doses than recommended, it could be quickly absorbed into the circulation in high amounts allowing rapid delivery to the heart and the potential for an overdose, similar to what can happen with overdosing with oral caffeine.

Furthermore, Revvies manufactures a caffeinated mouth strip claimed to deliver 40 mg caffeine/strip. Revvies advertises rapid caffeine delivery, as the strip dissolves on the tongue in just 30 s [70]. Lastly, Sprayable Energy claims to deliver 12.5 mg of caffeine/four sprays, and touts the benefit of sustained, slow release energy due to the prolonged absorption of caffeine through the skin [71]. However, there is no research to support these claims.

## Conclusions

Caffeine in chewing gum can be effectively administered at doses up to 200 mg, and higher with repeated dosing. Caffeine delivered via chewing gum is absorbed quicker through the buccal mucosa compared with capsule delivery and absorption in the gut, although total caffeine absorption over time is not different. Delivering caffeine in chewing gum improved endurance cycling performance, and there is limited evidence that repeated sprint cycling and power production are improved. Mouth rinsing with caffeine may stimulate nerves with direct links to the brain, in addition to any caffeine absorption that occurs in the mouth. However, caffeine mouth rinsing has not been shown to improve cognitive performance, although there is limited support for improvements in reaction time and cognitive control. It appears that delivering caffeine with mouth rinsing improved short-duration, high-intensity, repeated sprinting in normal and depleted glycogen states, while the majority of the literature indicated no ergogenic effect on aerobic exercise performance, and any effects on resistance exercise have not been adequately examined. Studies with caffeinated energy drinks have generally not examined the individual effects of caffeine on performance, as other documented (CHO) and potential (taurine) active ingredients are present. Caffeinated aerosol mouth and nasal sprays are gaining popularity as caffeine may stimulate nerves with direct brain connections and enter the blood via mucosal and pulmonary absorption. However, there is little support for any ergogenic effects as the delivery and/or effectiveness of caffeine delivered in this manner may be too small. Overall, direct measures of brain activation and entry of caffeine into the blood are generally limited or lacking when examining alternate forms of caffeine delivery in doses that are ≤ 200 mg. There is also a lack of research examining trained athletes and female subjects receiving alternate forms of caffeine delivery. Future research should also consider assessing the caffeine content of commercially available products prior to experimentation, as there may be a large variation in caffeine content within and between products.
